# PRODIGEN: visualizing the probability landscape of stochastic gene regulatory networks in state and time space

**DOI:** 10.1186/s12859-016-1447-1

**Published:** 2017-02-15

**Authors:** Chihua Ma, Timothy Luciani, Anna Terebus, Jie Liang, G. Elisabeta Marai

**Affiliations:** 1 Electronic Visualization Laboratory, Department of Computer Science, University of Illinois at Chicago, 851 S. Morgan St (M/C 152), Room 1120 SEO, Chicago, 60607 IL US; 2 Department of Bioengineering, University of Illinois at Chicago, 851 S. Morgan St (M/C 063), Room 218 SEO, Chicago, 60607 IL USA

**Keywords:** Systems biology, Feature detection and tracking, Network analysis, Modelling/Simulation

## Abstract

**Background:**

Visualizing the complex probability landscape of stochastic gene regulatory networks can further biologists’ understanding of phenotypic behavior associated with specific genes.

**Results:**

We present PRODIGEN (PRObability DIstribution of GEne Networks), a web-based visual analysis tool for the systematic exploration of probability distributions over simulation time and state space in such networks. PRODIGEN was designed in collaboration with bioinformaticians who research stochastic gene networks. The analysis tool combines in a novel way existing, expanded, and new visual encodings to capture the time-varying characteristics of probability distributions: spaghetti plots over one dimensional projection, heatmaps of distributions over 2D projections, enhanced with overlaid time curves to display temporal changes, and novel individual glyphs of state information corresponding to particular peaks.

**Conclusions:**

We demonstrate the effectiveness of the tool through two case studies on the computed probabilistic landscape of a gene regulatory network and of a toggle-switch network. Domain expert feedback indicates that our visual approach can help biologists: 1) visualize probabilities of stable states, 2) explore the temporal probability distributions, and 3) discover small peaks in the probability landscape that have potential relation to specific diseases.

**Electronic supplementary material:**

The online version of this article (doi:10.1186/s12859-016-1447-1) contains supplementary material, which is available to authorized users.

## Background

Gene regulatory networks encode those interactions among genes and proteins that regulate cellular processes, such as the expression of messenger RNA (mRNA). These interactions dictate the expression levels of genes as well as the production of particular proteins, and thus play a critical role in regulating biological functions, from metabolism to cell differentiation. Such networks typically involve small copy numbers of the molecular species and large differences in the species reaction rates. Because of these factors, the network interactions have a stochastic nature [1–3], i.e. they are unpredictable through the influence of a random variable. Modeling the stochasticity of genetic circuits is an important field of research in systems biology, and can help elucidate the mechanisms of cell behavior, which in turn can be the basis of diseases. These models can further enable predictions of important phenotypic cellular states.

The computational study of stochastic properties in gene networks is, however, a challenging task. Ordinary and stochastic differential equations methods may be inadequate in computing accurately the dynamic and steady state probabilistic behavior of gene networks [4], while the Gillespie stochastic simulation algorithm [5] is exact, but can fail in capturing rare events [6–8]. However, the formulation of the discrete Chemical Master Equation (CME) can be used to analyze the dynamics and stochastic nature of gene regulatory networks with low copy number of species. The CME framework can allow, for example, for the dynamics and stochasticity of a network with small copy numbers of molecular species to be fully described by probability distributions in both the state space and time space.

However, the analysis of these probability distributions is difficult due to their spatiotemporal and multidimensional nature, and due to the typically large number of simulations run under varying settings. Moreover, stochastic network researchers often emphasize that what is of biological significance is often not of statistical significance — numerical analyses often miss small or rare events of particular biological relevance. A visual approach can help, in contrast, in mining the network dynamics through the landscape defined by these probability distributions. For example, visualizing the number, location, and behavior of probability peaks could indicate the number of stable states for a given network and set of model parameters. We note that the state of the art reports in stochastic network modeling only employ visualization post facto. Our collaborators indicate that once the researchers know what they are looking for, they typically use a plotting software like R or Matlab to generate explanatory projection images and animations of the peaks. However, no visual tools exist to support the exploratory analysis of simulated data.

In this paper, we introduce a web-based visual analysis tool for the exploration of time-varying probability landscapes over the state space in stochastic networks. PRODIGEN (PRObability DIstribution of GEne Networks) supports the exploration of probability distributions in both state and time space. PRODIGEN captures probability distributions with projections at multiple levels, such as spaghetti plots for one dimensional projection and enhanced heatmaps for two dimensional projection. The main contributions of this work are as follows:

- We provide a description of the domain data and tasks in stochastic biological network modeling and analysis.
- We propose several visual encodings to represent the probability landscape in multiple dimensions, including one dimension, two dimensions, and three dimensions.
- We implement an interactive web-based visual explorer, PRODIGEN, that combines these visual encodings to enable the exploration of probability distributions across both state and time.
- We evaluate the visualization system through case studies and report a summary of the feedback provided by domain experts.

## Related work

**Stochastic network modeling.** The discrete chemical master equation (dCME) provides a fundamental framework for studying stochasticity in molecular-level networks. Because of the multi-scale nature of many networks, directly solving dCMEs is intractable due to the exploding size of the state space. To address this limitation, the ACME (Accurate Chemical Master Equation) algorithm [9, 10] was introduced as an optimal algorithm for the exhaustive enumeration of discrete microstates. The algorithm is based on the decomposition of stochastic reaction network into multiple independent components called buffer queues, each governed by its own birth-death process. The approach has the advantage of more effective usage of the overall finite state space, rapid estimation of errors, and estimation of required buffer size in order to maintain pre-defined error tolerance.

Several systems have been tested with the ACME method, which we also employ in this work. The steady state and time-evolving behavior has been previously computed for several biologically important networks, including the genetic toggle switch model, the phage-lambda epigenetic circuit, and the 16-node MAPK cascade [9–11]. The same method has been used for modeling important stochastic network motifs such as Single Input and Coupled Toggle Switch Modules [12].

**Probability distribution visualization.** Currently, there are only a few systematic ways of visualizing probability distributions at every location and time. The distribution data in 2D space is typically encoded by a 2D color map. Kao et al. [13, 14] present a number of methods for visualizing 2D probability distributions. They color a scale field to provide an overall impression of distribution data sets over a 2D spatial domain. Luo et al. [15] extend existing visualization methods, such as pseudocolor, to support such distribution data. Potter et al. [16] present a visualization system called ProbVis for exploring differences between distributions across a spatial domain. They also use a color map to encode the distance measure. 3D projections suffer however from overlap problems. To circumvent this problem, in this work we color-encode probability values on a 2D map, respectively as height.

**Heatmaps in biostatistics.** Heatmaps have been traditionally used to display statistical data, from gene expression and metabolomics to urban and network evolution data [17–20]. In general, heatmaps are used to describe the variables which can be considered as a function of two inputs represented by the rows and columns. We use similar heatmaps representations, in which both rows and columns represent the location in each dimension, and the cell shows the probability value at a particular location in two dimensions.

**Spaghetti plots in ensemble visualization.** The computation process used in this biology problem generates spatial distribution datasets across multiple time steps. By extension, these datasets can be regarded as ensemble data — a collection of multiple related but different datasets, such as simulations in general. Several techniques have been proposed for ensemble visualization. Spaghetti plots, overlaying plots of individual ensemble members, are a well-known technique for visualizing system flows, including flows in biology, medicine, and meteorology. Obermaier and Joy [21] state that spaghetti plots, as a feature-based visualization, provide an overall impression of the whole ensembles and allow comparisons between ensemble members. Luo et al. [15] present the use of spaghetti plots in meteorology. Potter et al. present Ensemble-Vis [22], a framework to support the visual analysis of ensemble space data. Sanyal et al. propose Noodles [23], a coordinated-view visualization tool, for visualizing weather ensemble uncertainty. In addition to novel uncertainty visualization techniques, they implement spaghetti plots for observing the model uncertainty. Wu and Zhang [24] introduce spaghetti plots for visualizing ensemble uncertainty. Ferstl et al. [25] present a new approach that extends spaghetti plots to extracted ensemble flows. Similar to these works, we adopt spaghetti plots, but in a new context.

## Methods

In this work we follow the nested visualization design model [26, 27], beginning with the domain characterization step.

### Data and task analysis

#### Stochastic network modeling

The study of stochastic gene regulatory networks is a challenging topic, as the system modeled may be large in both the state space and time. Recent developments of the ACME method [9, 10] enable reduction of the state space from *O*(*b* ^*n*^) to $\left (\prod _{j}{b + n_{j} \choose n_{j}}\right)$, $\sum _{j=1}^{m} n_{j} = n$, where *n* is the number of species, *m* is the number of Molecular Equivalence Groups (MEGs)— the number of molecular species subgroups in the network, such that member species of a subgroup can be transformed into each other through one or more mass-balanced reactions, *n*_*j*_ is the number of species belonging to group MEG _*j*_, and *b* is the maximum number of molecules in MEG _*j*_. A buffer of size *b* is assigned to each of the MEGs involved in the model. These developments allow optimal enumeration of the state space for the system.

We briefly discuss the state space over which we visualize the probability landscape. Assume a system contains *n* molecular species *x*_*i*_, and *m* buffers of size *C*(*x*_*j*_). The ACME method assigns a buffer of size *C*(*x*_*j*_) to MEG _*j*_. The state space size is then equal to the product of *m* combinations of ${C(x_{1}) + n_{1} \choose n_{1}} \times {C(x_{2}) + n_{2} \choose n_{2}} \times \dots \times {C(x_{m}) + n_{m} \choose n_{m}}$, where × denotes a simple multiplication.

A *microstate* *s*_*k*_ of the system, where *k*∈(1,*N*), is defined by the combination of *numbers* of molecules (copy numbers) of every *species* (*n*_*k*_(*x*_1_), *n*_*k*_(*x*_2_),… *n*_*k*_(*x*_*n*_)). The *state space* is formed by all the possible microstates that the system can visit from a given initial condition. The *state space table* (Table 1) displays a microstate as a single row, for a total of *N* microstates.

**Table 1:** The state space of a system with *n* molecular species *x*_*j*_ and *N* microstates *s*_*i*_. *n*_*i*_(*x*_*j*_) denotes the copy number of molecular species *x*_*j*_ at state *s*_*i*_

Copy number		Molecule species			
		*x*_1_	*x*_2_	…	*x*_*n*_
States	*s*_1_	*n*_1_(*x*_1_)	*n*_1_(*x*_2_)	…	*n*_1_(*x*_*n*_)
	*s*_2_	*n*_2_(*x*_1_)	*n*_2_(*x*_2_)	…	*n*_2_(*x*_*n*_)
	…	…	…	…	…
	*s*_*N*_	*n*_*N*_(*x*_1_)	*n*_*N*_(*x*_2_)	…	*n*_*N*_(*x*_*n*_)

Each row of the *probability matrix* shown in Table 2 contains the probability values of the corresponding state (row) in the state space table (Table 1) over time. *P*_*ij*_ represents the probability value of state *s*_*i*_ at time *t*_*j*_, and *P*_*ij*_ is a float value between 0 and 1. The sum of the probabilities of all the microstates at any particular time is equal to 1.

**Table 2:** The probability matrix displays the probability distributions over *N* microstates across *T* time steps

	*t*_1_	t_2_	…	*t*_*T*_
*s*_1_	*p*_11_	*p*_12_	…	*p*_1*T*_
*s*_2_	*p*_21_	*p*_22_	…	*p*_2*T*_
…	…	…	…	…
*s*_*N*_	*p*_*N*1_	*p*_*N*2_	…	*p*_*NT*_
Sum	1	1	1	1

The combination of data stored in Tables 1 and 2 represents one *simulation* of a system that consists of *m* molecular species with the state space size of *N* across *T* time steps.

In this multidimensional probability space, the biology researchers are interested in identifying both *global and local probability peaks*. Global peaks are in general easily detected. However, local peaks are difficult to notice, when they exist, because they have low probability values. The number of peaks and their probability values can be computed analytically; however, analyzing the locations of peaks requires visualization.

The domain experts have been working with systems that may contain at most 16 molecular species, but over 1 million microstates across thousands of time steps. A system may feature tens of parameter settings, which in turn produce tens of simulations. In this work we use two stochastic network models: a toggle switch system and a transcription regulation network. The toggle switch system features two genes, each expressing one protein, with 120 respectively 240 maximum copy number. The transcription network contains three genes which express each one protein. Both proteins and genes (DNA molecules) are reactants in the reaction systems of biomolecules.

#### Task analysis

Through multiple interviews and observation sessions with the domain experts, we identified a set of four task groups related to the exploration of the time-varying probability landscape in a stochastic system. Given a simulation run of a stochastic gene regulatory network, the identified tasks are as follows:

- T1: Display the overall plots of probability distributions at multiple dimensional levels: for example, for each molecular species in one dimensional projection and for the combination of any two molecular species in two dimensional projection.
- T2: Discover peaks in multiple dimensional projections. Identify the number of peaks and their probability values. Observe and inspect *small, local* peaks.
- T3: Identify the locations of peaks at multiple dimensional levels. The “location” denotes a collection of corresponding states in the state space.
- T4: Track temporal changes of the system. Observe the probability landscape changes over time, including the number of peaks and their locations.

Based on the visual data analysis taxonomy [27], we match these task groups with four taxonomy categories: Present: T1, T4, Discover: T2, T3, Explore: T2, T4, and Identify: T2, T3.

In addition to the functional requirements outline above, we also identified nonfunctional requirements, such as scalability of the system, learnability, and availability of the system on the web.

#### Data acquisition and preprocessing

The simulation results from domain experts are stored in groups of text files: one state space text file in the format shown in Table 1, and *T* (time) probability files. Each probability text file, shown by each column in Table 2, encodes the probability distribution over the state space at a particular time step. The size of a single probability file in a system with a state space size of 680430 is roughly 18 MB.

Because the probability distributions can be high-dimensional while still having a spatial distribution, we preprocess the data through dimensionality reduction to a lower space which can be used for visualization. Furthermore, due to the importance of peaks to the user tasks, we explicitly compute and detect these structures. Due to the large-scale of the data, we pre-process the data offline when reducing the dimension of the state space for visualization. In contrast, peak detection is computed online.

**Dimensionality reduction.** Because the probability distributions of stochastic networks can be potentially defined over a space higher than 2 dimensions, we explore dimensionality reduction via aggregation and projection. To map the data visually, we compute projections to one dimension and to two dimensions, which is commonly done and accepted in the target domain; an additional projection to three dimensions and visualization using volume rendering was attempted and discarded during the prototyping stage.

To project the landscape defined by the probability distributions to one dimension, we aggregate the microstates with the same copy number of a particular protein to form a new state space in one dimension. We repeat the aggregation process for each species of proteins. For example, in the 2-gene toggle switch system, we obtain two probability distributions over two 1D state spaces: one for protein A and the other for protein B.

To project the probability landscape to two dimensions, we aggregate the microstates for which the combinations of the copy numbers of any two proteins are the same. For example, consider a 3-protein example with only two states possible in the case of 0 copies of protein 1 and 0 proteins for protein 2: state (0,0,0) of probability 0.05 and state (0,0,1) of probability 0.05. The new aggregated state (0,0) will have probability 0.1. We repeat the process for all possible combinations of any two proteins in order to obtain multiple 2D state spaces. For example, the 2-gene toggle switch network only has one 2D state space, while a regulatory gene network with three genes consists of three state spaces projected to 2D, and a network with four genes would include six 2D state spaces.

**Peak detection.** To detect the peaks, we use a gradient estimate approach. We compare all the states to their neighbors, as projected to one dimension. If a state has higher probability value than both its neighbors and its value is above a threshold of 1e-12 (empirically determined based on the simulation threshold for error), the state is defined as a peak. Since the probability distribution along each species dimension is independent, the number of peaks in the entire system is multiplied by the numbers of peaks in each dimension. The peak locations in the system are all the combinations of species locations. The process of peak detection is completed online.

### Visual design

We have designed the visual analysis tools through a parallel prototyping approach, during which multiple low to mid-fidelity prototypes were sketched in parallel, and presented for feedback to the domain experts. The domain experts are particularly attuned to surface plots of probability distributions, as indicated by their use of “landscape” terminology. However, such plots do not capture directly temporal features: time series of 3D surface plots are usually generated as a movie to display the temporal changes. Thus, visual representations with temporal features embedded were of particular interest. As part of this process, we explored multiple potential visual designs, including star plots, small multiples, animation, space-time-cubes, volume renderings and variations of topographic maps, and converged towards those encodings which best preserved features of interest (such as peaks and temporal behavior) while avoiding occlusion.

**Fig. 1:**
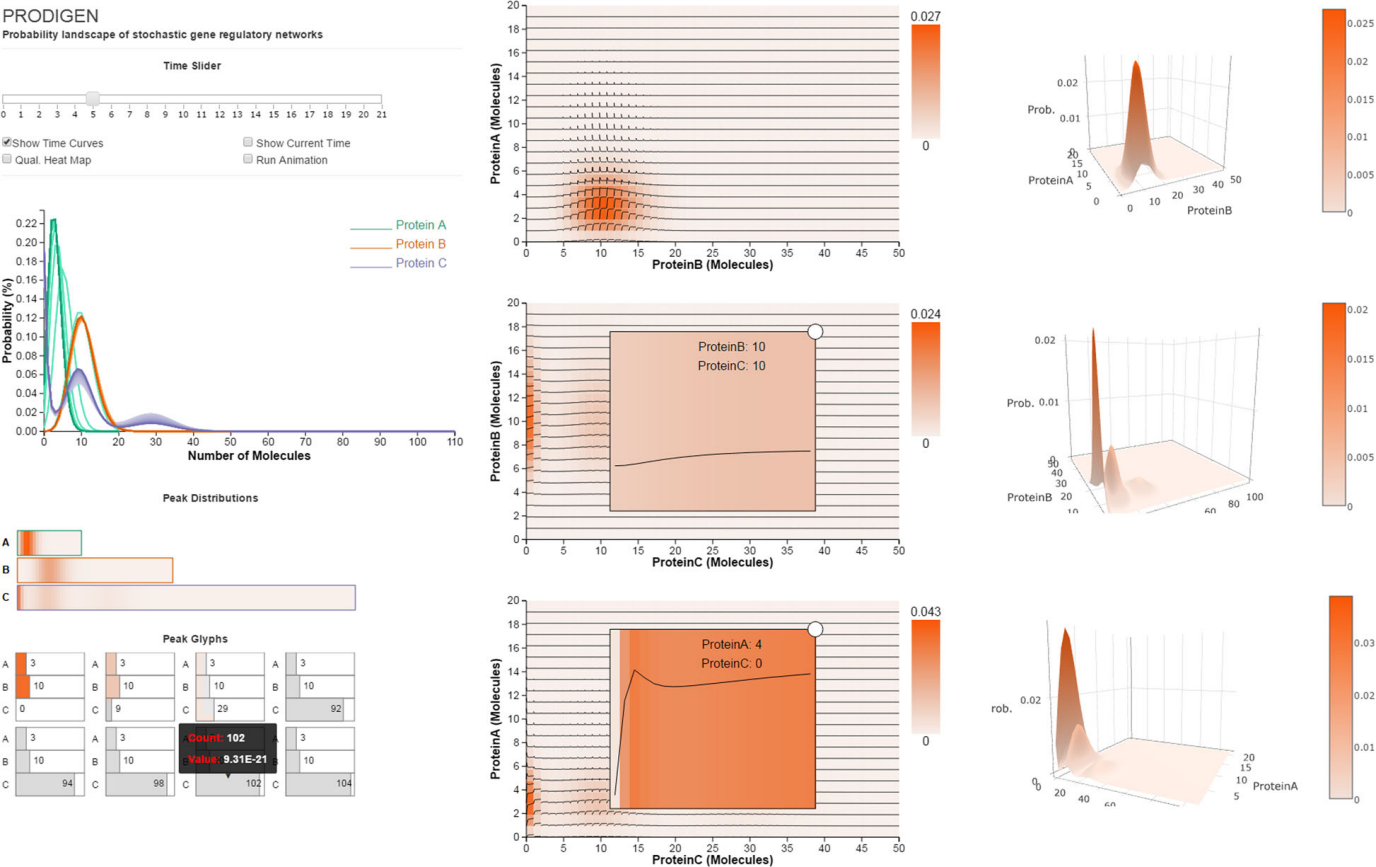
The PRODIGEN interface consists of several visual components: (*left top*) the ensemble Spaghetti Plots view shows the probability distribution over time, for all the proteins in a system; (*left middle*) the 1D heatmap view shows a per-protein view of the probability peaks; (*left bottom*) the Peak Glyph view (displayed as a small multiple) represents all the probability peak states in the system; (*center*) the 2D heatmap view is enhanced with time-curves, and shows the probability peak correlation between protein pairs over space and time; (*right*) the animated 3D Surface view describes the shapes of peaks over space and time

The final prototype of PRODIGEN (Fig. 1) consists of a multi-view design with several visual components: 1) a spaghetti plot view that shows the temporal changes of probability values for every gene; 2) a small multiple 1D heatmap view; 3) a small multiple enhanced 2D heatmap view which displays the probability distributions over the state space projected to two dimensions; 4) a peak glyph view which represents the corresponding states of all the probability peaks detected in the system; and 5) a small multiple 3D surface view. The information shown in the heatmaps and peak glyphs changes according to the user-selected time step, as the user drags a time slider. We describe in detail each visual component below.

#### Ensemble spaghetti plot view

Spaghetti plots have been traditionally used to visualize ensemble data; each single plot represents an individual ensemble member. Color-coding may also be used to differentiate members. In this work, we extend the spaghetti concept to specific simulations over time: by extension, each “ensemble” member represents the probability behavior at a particular time point. The horizontal axis represents the copy numbers of molecules, while the vertical axis represents the probability value. Thus, an individual plot describes the probability distribution over the states with the copy number from zero to the maximum copy number for that protein.

We use color to encode different species of proteins, and the intensity of the color to encode the probability distribution of the corresponding protein at different time steps. The plot intensity from lighter to darker represents the time from the beginning of the simulation to the end.

In our early prototyping stage a third dimension was used to encode time, in the style of space-time cube representations [28]. In practice, however, the encoding suffered from occlusions which made difficult the tracking of temporal peak changes, and was later discarded. The domain experts specifically stated that the 2D ensemble spaghetti plots yielded better performance than the cube representations.

**Fig. 2:**
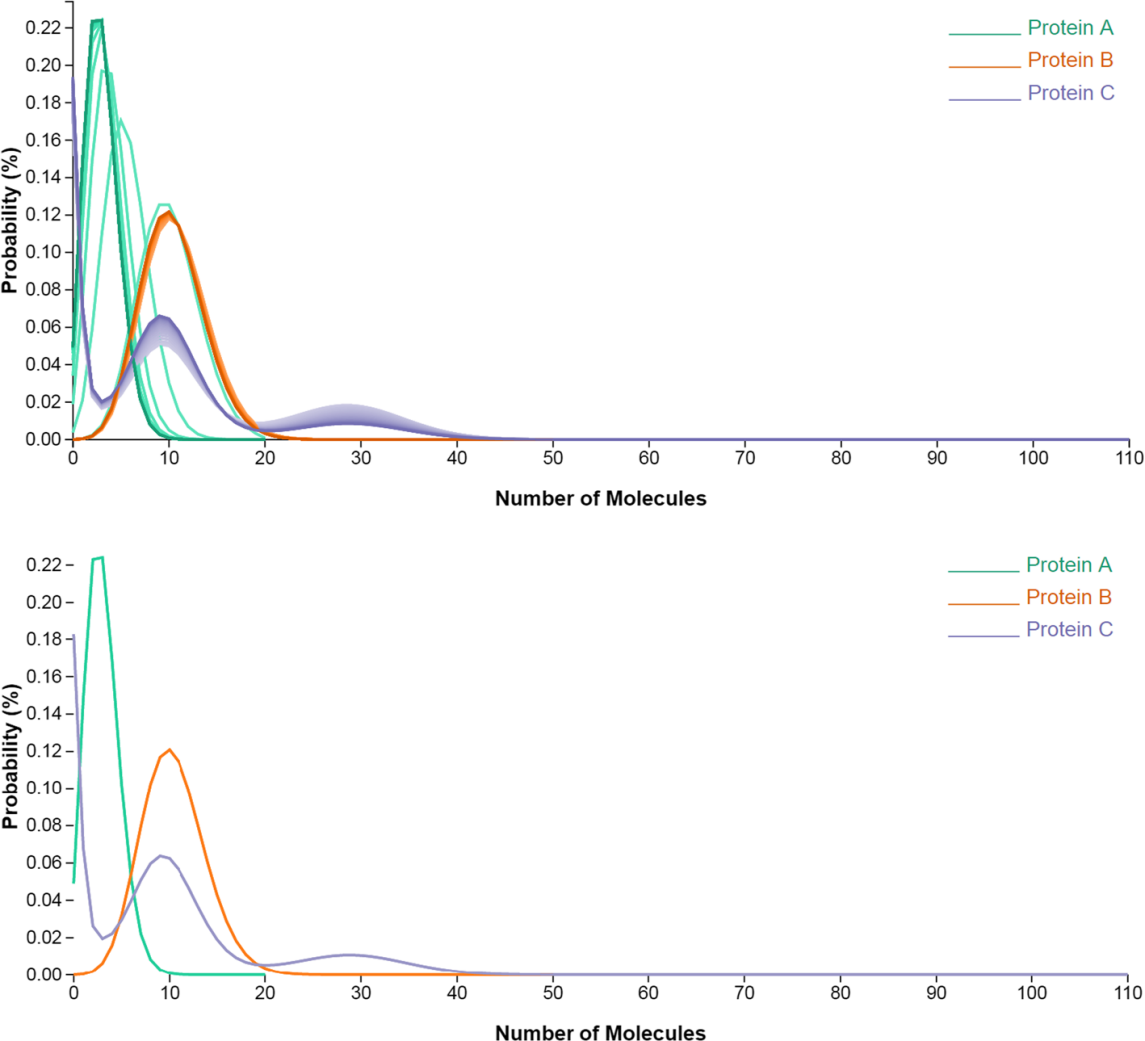
Spaghetti Plots (*top*) show the probability distribution of each gene and the changes in distributions over time. The Spaghetti Plots view on the *bottom* displays the probability distribution of each gene at a user selected time step

In Fig. 2 (top), the Protein A of spaghetti plots represents the temporal probability distribution of protein A. In this representation, the peak changes can be easily tracked in terms of both peak location and value. We notice that protein A has only one peak, whose location shifts to the left in time towards the state with a lower copy number, and whose probability value increases over time. Protein B also has one peak, which stays at the same location without too much change in the probability value. Protein C has three peaks. The locations of these three peaks do not change over time. However, the probability value of one peak in the middle increases as the other one on the right decreases over time.

In most cases, the peaks either increase or decrease over time, and thus do not cause overlaying or crossing issues. Rare plot overlays and crossings bear in fact meaning, by encoding frequent peak location changes (see Protein A in Fig. 2) or peak changes in both directions (increasing and decreasing).

However, it is not easy to detect the probability distribution at a particular time step from the spaghetti plots. To this end, a checkbox filter allows the users to draw the plots at an interactively-selected time step. Figure 2 (bottom) displays the probability distributions of these three proteins at the 10th time step.

#### Enhanced heatmaps

**Fig. 3:**
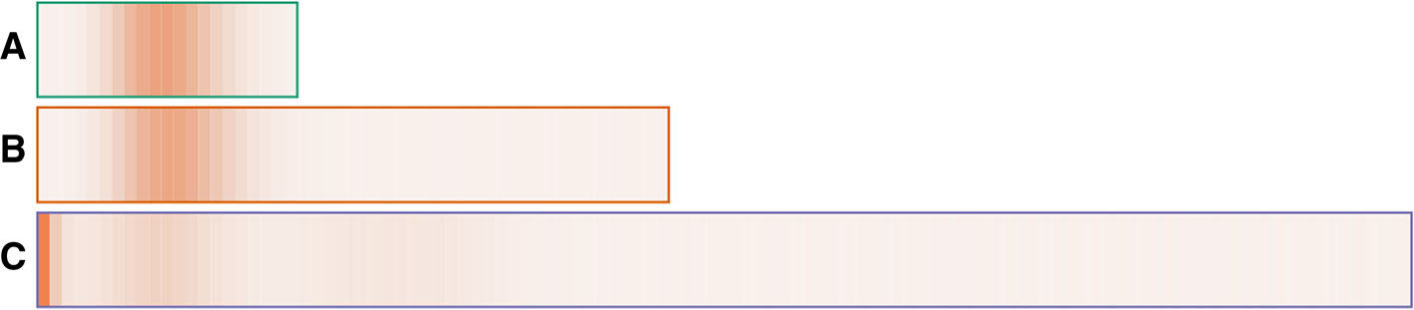
Three 1D heatmaps represent probability distributions projected to one dimension of three proteins **a**, **b** and **c**. **a** and **b** have one peak each, while **c** has three peaks

To show the lower-dimensionality projection of probability landscapes we chose as a basis a heatmap encoding, due to their efficiency in visualizing distribution data. Our domain experts, like most bioinformaticians, were familiar with heatmap representations, compared to other visual representations. We employ both one-dimensional heatmaps and an enhanced version of two-dimensional heatmaps, displayed each time in a small multiples view. The spaghetti view uses a categorical color scheme for the different species, while the aggregated views described below employ a non-overlapping qualitative color scheme to encode probability. The 1-dimensional heatmaps described below (Fig. 3) help bridge the two color schemes. The domain experts did not report or experience during testing any issues related to this dual use of color intensity.

**1D heatmaps.** 1D heatmaps display the probability distribution over the state space projected to one dimensional space. The 1D state space is represented by the horizontal direction in the heatmap. The color intensity of each state represents its aggregated probability value. The heatmaps are stacked up vertically, and each heatmap has a border color-linked to the spaghetti plot for that protein.

Figure 3 shows the probability distribution over one dimensional state space for three proteins at time 0. When projecting to Pa which represents protein A, the only probability peak is located mid-axis, which corresponds to the state in which the copy number of molecule Pa is 10, half of the maximum copy number. Similarly, the peak in the Pb (protein B) projection is also at the state with the copy number of 10, and these two peaks have similar probability values, as indicated by their similar intensities. However, in the Pc (protein C) projection, there are three peaks: a large peak leftmost along the axis (where the state has a copy number of 0), one small peak nine states to the right, and one much smaller peak twenty nine states further right. The smallest peak in the Pc projection may not be easily observed from the heatmaps, but can be quickly detected through the peak glyphs that will be explained below.

**Fig. 4:**
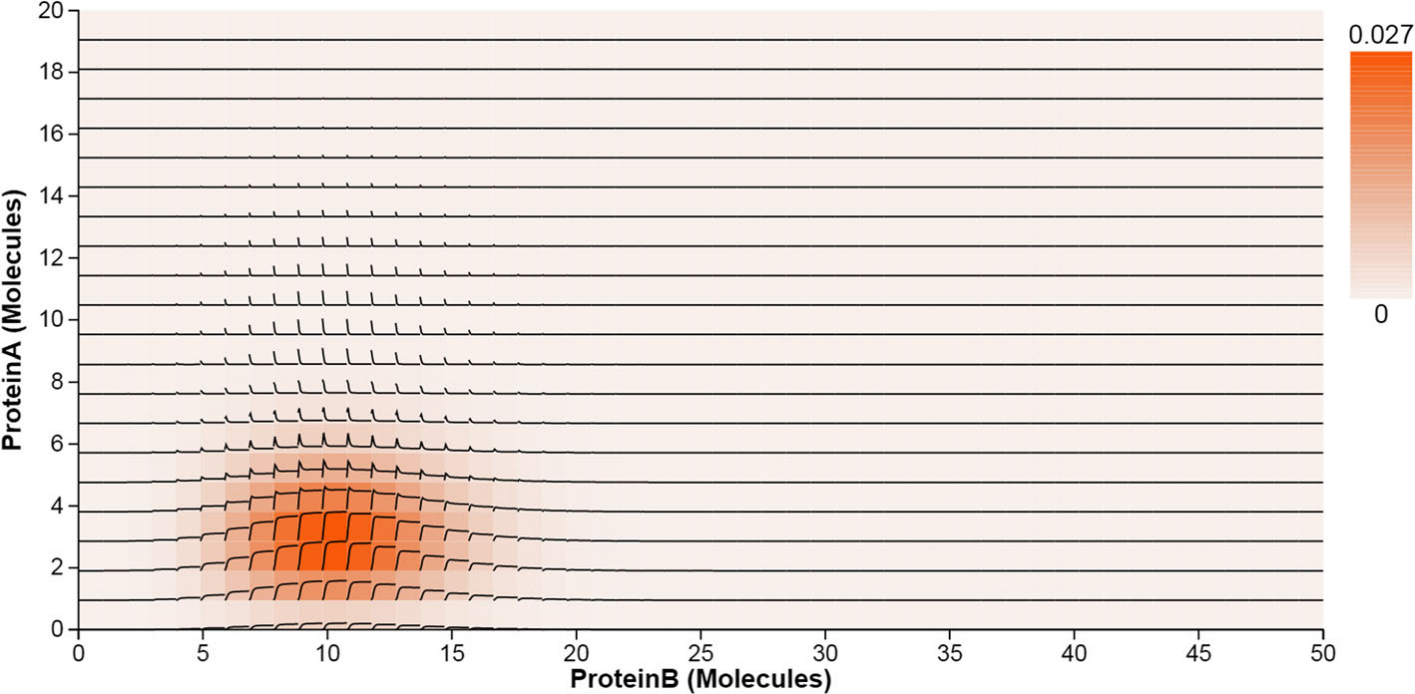
The 2D heatmap displays the probability distribution over the 2D state space projected for the pair of Pa and Pb. Time *Curves* overlaid on the 2D heatmap indicate how the probability values in the 2D state space change over time

**Fig. 5:**
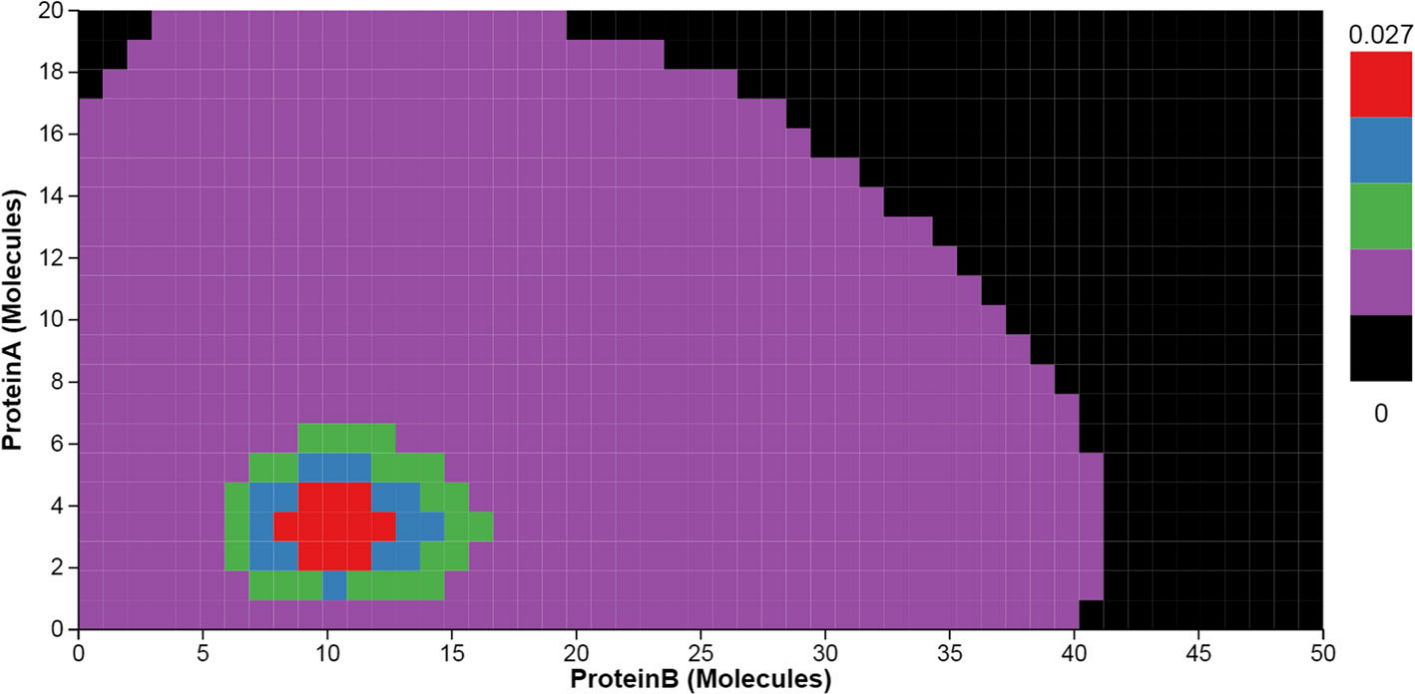
2D heatmaps using a qualitative colormap

**2D heatmaps.** Similar to 1D heatmaps, 2D heatmaps display the probability distribution over the state space, but projected to two dimensional space. The horizontal and vertical axes represent the copy numbers of molecules for any two protein species in the system. The cell at the intersection of two molecule copy numbers represents the state that aggregates the microstates with those copy numbers. The intensity of the cell encodes the probability value. Figure 4 indicates there is only one peak located at (3, 10) in the 2D state space projected to Pa and Pb. The 2D heatmaps are arranged in a small multiple display, using identical axes mappings to support comparison of the peaks. Per our collaborators’ request, the heatmap can also be colored using a qualitative colormap (Fig. 5), similar to the rainbow maps they had used before in numerical software packages; the qualitative map is derived from ColorBrewer (colorbrewer2.org) and allows the users to highlight different peak classes and thus presumably better identify small peaks.

**Overlaid time curves.** Heatmaps are able to provide an overview of the probability distributions over the state space in one and two dimensions. However, it is hard to track temporal changes from this aggregated visual representation. Since the changes in probability values over time can be displayed as a line plot, we create an extended heatmap representation by overlaying such line plots on each cell (state) in the 2D heatmaps. The time curves show the dynamics of the system, which include the changes in probability values and peak locations over time. A flat curve means no obvious change, while a steep curve means a big change in the probability value. The user can check the time-curves checkbox to display these curves.

In Fig. 4, two groups of steep curves indicate that the peak in the state space projected to Pa and Pb moves in the direction in which Pa has lower copies at the early time, and stays there during the rest of the simulation time. The probability value of the peak increases as the location of the peak changes.

**Fig. 6:**
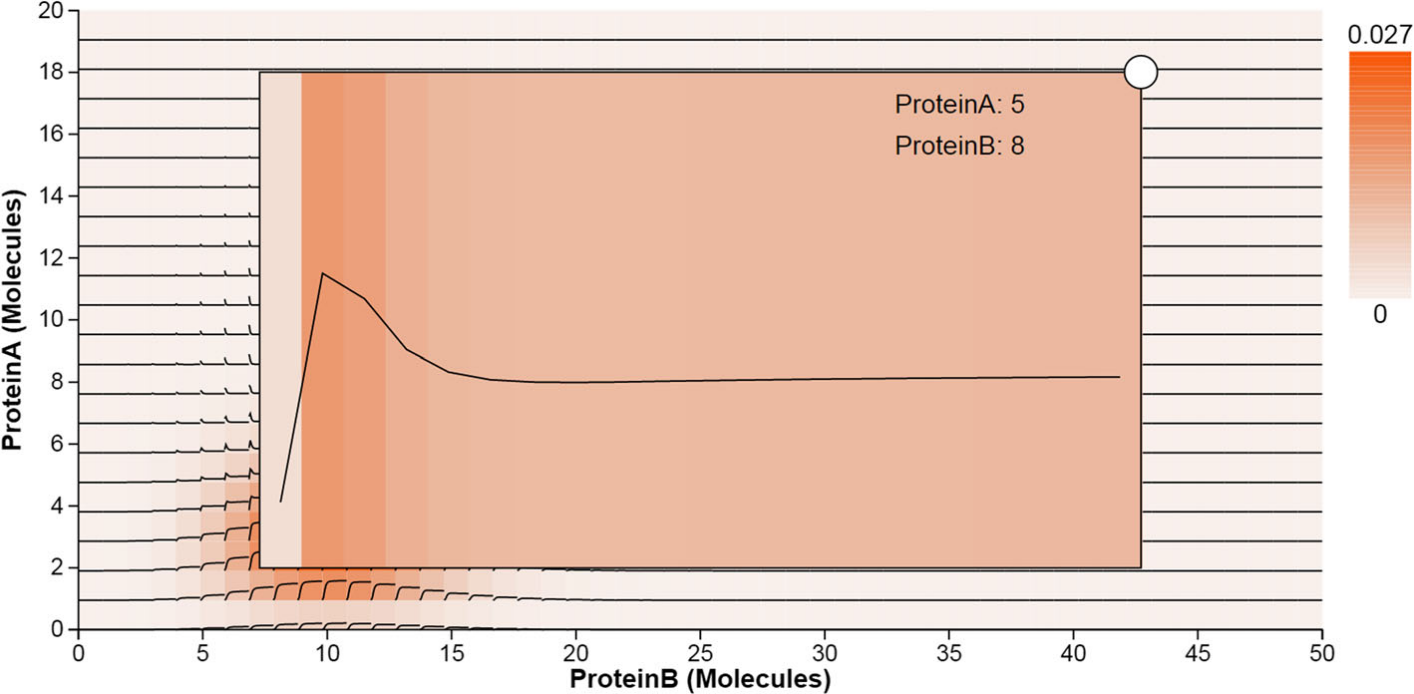
Probability temporal distribution on a user selected state in which Pa has five copies of molecules and Pb has eight

**Details-on-demand.** Since the 2D heatmap can display state spaces that contain thousands of states, it may be difficult to observe how the probability of a specific state changes over time. To support detailed inspection of a particular state, we enlarge on demand the size of the cell corresponding to a user-selected state. When the user clicks on an interesting cell, a detail window is overlaid, showing the time curve for that state. Figure 6 is an example of the detail view of the state (5, 8), in which Pa has 5 molecule copies and Pb has 8 copies. We notice that there is a steep and large increase in the probability value during the first two time steps. Later, the probability decreases in the following four time steps, and keeps roughly constant for the rest of time.

#### Peak glyphs

Although the heatmaps view can provide an overview which indicates the location of peaks, users often wish to identify the location accurately. Furthermore, the heatmap representations can make small peak detection difficult. To indicate the peak location, we have experimented with a number of existing multidimensional encodings, including star plots. However, since a stochastic gene system consists of a large number of microstates and each microstate is represented by the combination of copy numbers of multiple molecular species at different scales, existing encodings failed to provide the location information of states in the system.

**Fig. 7:**
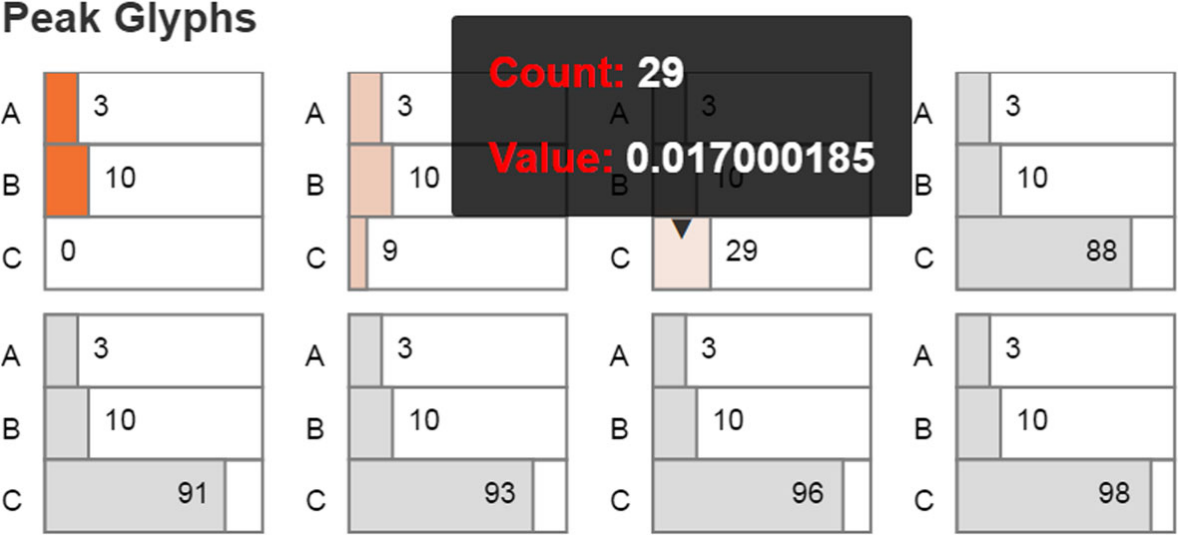
A small multiple of Peak Glyphs with eight peak states, out of which only three are authentic peaks. The remaining five states, shown in *gray*, were identified as computational errors

Instead, the end-result of the prototyping phase was a glyph to display the detailed information of a peak state, such as the combination of copy numbers. The peak glyph is composed of a stack of horizontal bars filled with darker or lighter intensities according to the probability value (Fig. 7). The number of bars represents the number of proteins, while the length of each bar represents the number of molecule copies of the corresponding protein. When displayed as a small multiple, the peak glyphs show all the peaks in the system. Because very small probability peaks are typically due to numerical error in the simulation, suspect peaks are displayed in gray. The view is controlled by the user selected timestep. Hovering over a glyph displays the detailed information about the peak.

Figure 7 displays eight potential peak states in a 3-protein system. However, five of these states appear as peaks likely due to numerical error and are shown in gray. Only three of the states are authentic peak states, at the location of (3, 10, 0), (3, 10, 9) and (3, 10, 29). The details on demand indicate, for example, that Pc has a peak at the state with 29 copies and that its peak value at that location is about 0.017. In general, the peak glyphs can be used as a detailed guide through the peak set generated by a system.

#### 3D surface view

**Fig. 8:**
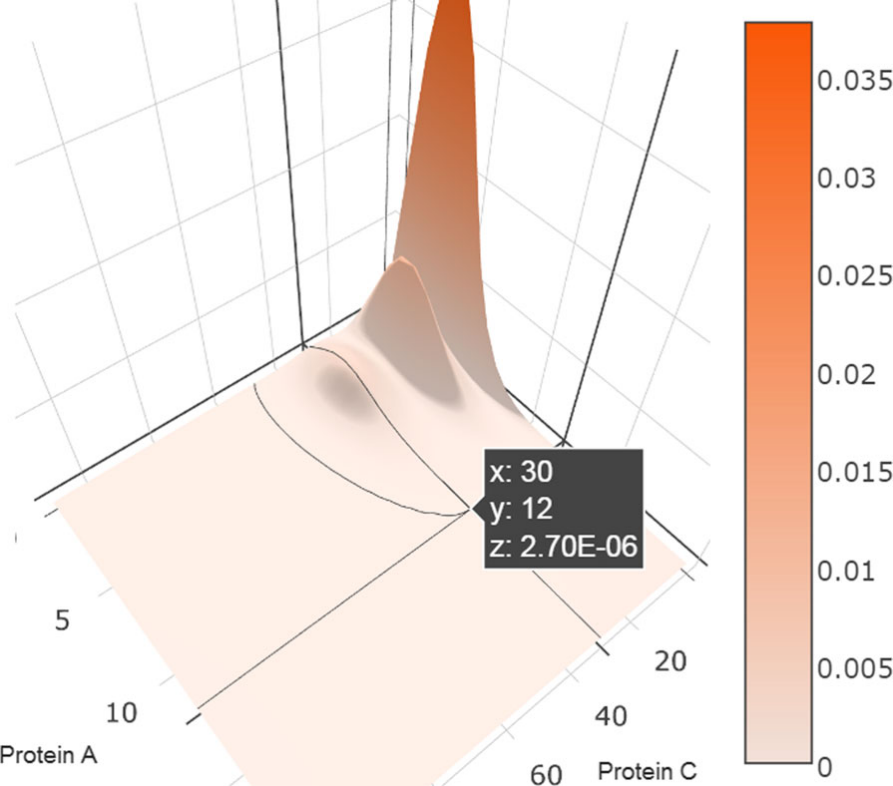
A 3D Surface Plot displays the probability distribution over the state space projected to **a** and **c**. Three peaks are distinguishable

Similar to the 2D heatmaps, the 3D surface plots also display the probability distributions over the 2D state space, but represent the probability value as height (z-axis) instead of encoding it by the color intensity (Fig. 8). As indicated above, the domain experts are particularly receptive to 3D surface plots, which reflect their understanding of probabilistic landscapes. In contrast, the complementary visual encodings were novel to our domain experts, and thus benefited from scaffolding through the familiar surface encodings. Compared to the 2D heatmaps, the advantage is that the surface plots can show the shapes of peaks in a manner similar to existing representations of probability landscapes. Both representations, extended heatmaps and surfaces, capture well the extent of peaks and the relative location of peaks with respect to one another.

We use the library Plotly.js for plotting the 3D surface of probability distribution; the library allows users to rotate and zoom in/out the surfaces. Hovering over the surface displays the location of the state and its probability value. We implement an animation option for playing the probability distributions across the entire time period as a movie, in which the dynamics of peaks including numbers, locations, relationships, values, and shapes can be easily detected. Similar to the heatmaps, the 3D surface plots are also displayed as a small multiple across the potentially multiple projections.

PRODIGEN is implemented as a web-based tool in JavaScript and uses the data visualization libraries D3 and Plotly.js.

## Results

We evaluate the effectiveness of PRODIGEN through two case studies completed with two experts, who are co-authors on this manuscript. One expert is a senior bioinformatics researcher with over thirty years of experience in the field, and the other is a junior researcher in bioinformatics who specializes in stochastic gene network modeling.

These two case studies demonstrate how our visual approach can assist domain experts in the exploration of probability landscapes in stochastic gene networks, and allows them to track the temporal changes as well. The first case study is an exploration of a transcription regulation network with three genes, and the second one studies a toggle switch system with two genes.

### Case study I: transcription regulation network

We illustrate the performance of PRODIGEN using the example of a transcription regulation network. A detailed description of the model architecture will be published by our collaborators. The system consists of three genes GeneA, GeneB, and GeneC, which express ProteinA, ProteinB, and ProteinC, correspondingly. C is the output of the system, and it is regulated by both B and A. Transcription regulations motifs such as this are common in studies of biological systems as, for example, *E. coli* [29], yeast [30], and mammals [31]. Their detailed modeling in principle can answer important biological questions such as the existence of multiple phenotypes corresponding to different numbers of stable states, adaptability of the systems to the change in one of the components [32]. These phenomena may be relevant for the study of important biological events, such as differentiation of the cell to a new cell type, tumor suppression, HIV latency versus replication, and cell adaptation to a stress. In this simulation, the domain expert decreased the expression level of A by a factor of approximately 3. The goal of the visual analysis was to observe how C responds to this change in the expression level of A.

The spaghetti plot in Fig. 1 (left middle) indicates that even a significantly decreased level of A does not affect significantly the behavior of the system. The spaghetti view clearly shows that A has one peak state, B has also one peak state, while C, which is regulated by both A and B, has three peak states. As simulation time advances, the peak-state corresponding to A shifts towards the left along the horizontal axis as the expression level of A decreases. In the same spaghetti plot, the peak corresponding to B is very stable; its clear signature in the plot shows very little change in either location or probability value. The three peaks of C also stay constantly in the same locations, and show little change in probability values over time. The spaghetti plot can be used to detect the number of peaks (T2), and track their temporal changes in one dimension (T4).

The 1-dimensional set of projections (Fig. 1 left) captures, as expected, the same number of peaks. The smaller and fainter peaks in the C projection caught the interest of the investigators, who remarked again on the biological significance of small peaks. Small peaks are important since they can correspond to a diseased state of the cell, which could be non-prevalent or rare in the organism and as such have a small number of molecules. It was easier to detect small peaks in the one dimensional view than in 2D, because in 2D the information gets distributed over a larger number of states. The value and location of the peaks are represented in both the spaghetti and 1-dimensional view in a qualitative way.

A step further, the peak glyph view (Fig. 1 left bottom) captures the accurate locations and probability values of the peak states (T2 and T3). The peaks with different values at different locations can correspond to different physiological conditions of the cell, e.g., healthy vs. diseased state or pluripotent vs. differentiated in stem cells. The gray glyphs show the pseudo peaks that are due to numerical errors. As simulation time advanced, the experts noticed an increase in numerical errors. With this observation in mind, the users moved back to exploring relatively more complex visual representations, such as the 2D heatmaps overlaid with the time curves and the 3D surface plots.

Next, we investigate the probability distributions over the entire state space of the transcription regulatory network (Fig. 1 center), and again focus on the same small peak. The distributions are displayed through three 2D heatmaps which are the projections of the three possible combinations of the original three components. The time curves overlaid on the heatmaps further capture the trace of peak movements (T4). For example, based on the shapes of time curves, we can conclude that the peak moves down in the heatmap projected to A and B. Last but not least, the surface plot animation (Fig. 1 right) confirms the smooth trajectory of the peaks. The 2D enhanced heatmap outperform other visual designs when the user focuses on tracking the temporal changes of probability distributions in 2D state spaces over time.

In conclusion, while the steady state probability landscapes of ProteinA and ProteinB are monostable, ProteinC has three peaks at steady-state. The decrease of the expression level of ProteinA drives the evolution of the system to a new steady state, which has decreased level of ProteinA, with the expression patterns of ProteinB and ProteinC both altered.

### Case study II: toggle switch system

**Fig. 9:**
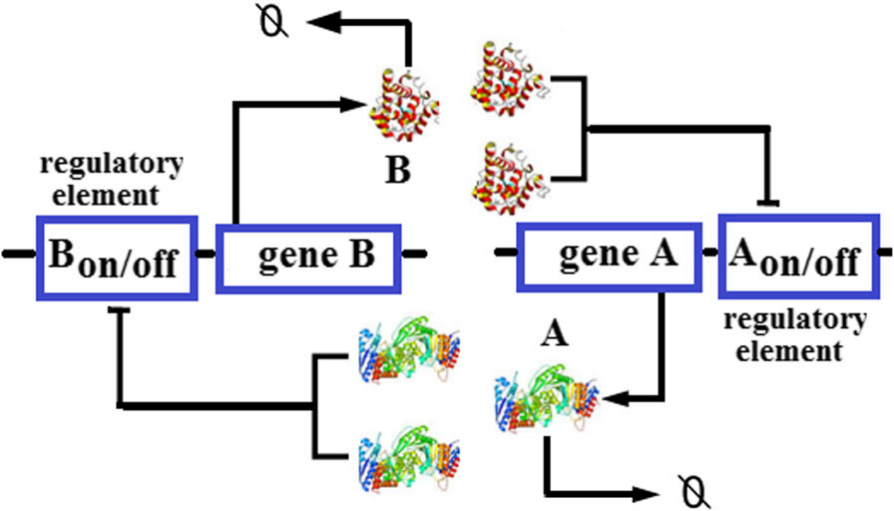
The genetic toggle switch network [33]

The stochastic network module we study here is a genetic toggle switch system consisting of two genes, A and B. Figure 9 shows the network topology. In this second model, single copies of gene A and gene B express protein products A and B. Gene A and gene B can repress the transcription of each other through binding the dimers of their protein products A and B on the promoter sites of the other gene B and A to form protein-DNA complexes [9, 10, 33]. Thus, this genetic network includes six molecular species, which are described in detail in Table 3. The genetic toggle switch network with two genes has four distinct stable states: “on-on” representing a state at which both gene A and gene B are unbound, “on-off” representing a state with unbound gene A and bound gene B, “off-on” for a state with bound gene A and unbound gene B, and “off-off” for a state with both bound gene A and gene B. In this work, the domain experts computed the state spaces under the initial condition of 0 copies of protein A and protein B, 1 copy of unbound gene A, 1 copy of unbound gene B, and 0 copies of bound gene A and bound gene B. By using the finite buffer method to solve the dCME of this system, we obtain a state space of size 115,200. After that, we directly compute the probability value of each state at each time point, which forms the probability landscape, and currently output for visualization the first 200 time steps.

**Table 3:** A descriptor of the 2-gene toggle switch network

Molecule name	Molecule structure	Max copy number
Pa	Protein A	120
Pb	Protein B	240
Da	GeneA in the unbound state	1
Db	GeneB in the unbound state	1
BDa	GeneA bound by protein B	1
BDb	GeneB bound by protein A	1

**Fig. 10:**
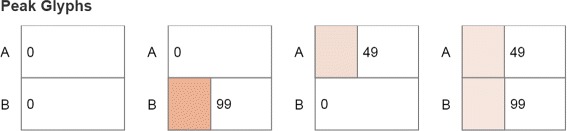
Peak glyphs for the toggle switch network showing four detected peaks and their exact coordinates

**Fig. 11:**
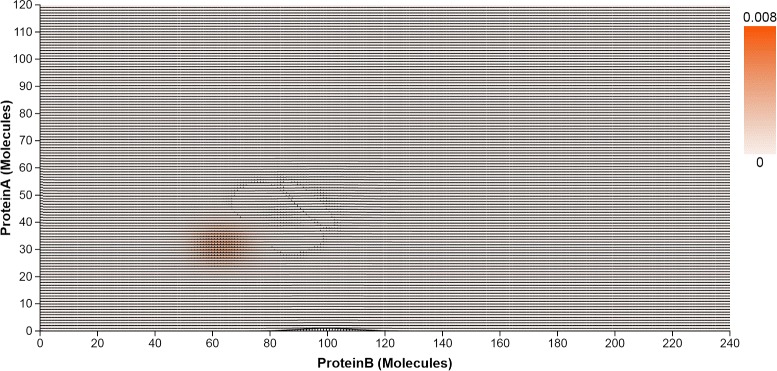
Toggle Switch 2D enhanced heatmap. The heatmap displays the probability distribution over the 2D state space projected for the pair of Pa and Pb. Time *Curves* overlaid on the 2D heatmap indicate how the probability values in the 2D state space change over time

**Fig. 12:**
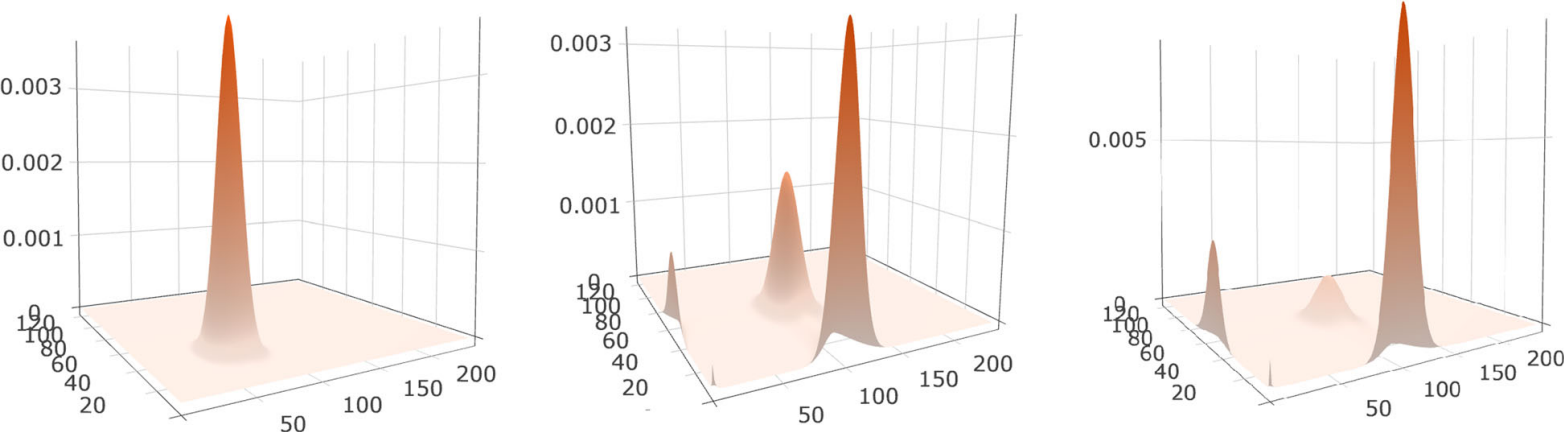
Probability landscape of the toggle switch system at three different time steps, showing four peaks; the least noticeable peak is located at (0,0)

The toggle switch system is known to have four peaks in the stable state, as captured by the peak glyphs (Fig. 10). However, at the early time, the system has only one peak, at the state (31, 63). The enhanced heatmaps (Fig. 11) and the animation offer an opportunity to observe how the peaks move in the state space in both the 2D heatmap and the 3D surface plot (T4). In addition to tracking the location changes, the surface plot shows efficiently the changes in probability values (Fig. 12). As simulation time advanced, we noticed three more peaks appear, as the early peak was getting smaller and transitioned to states with higher copy numbers of both protein species. Figure 12 displays the four peaks in the 3D surface plot at different time steps. Later on, the system becomes more stable. These peaks stay in a roughly fixed location with only small changes in the probability value. Thus, we can confirm that the system reaches a steady state.

The peak glyphs successfully identified the largest peak (T2). We see one of the three late peaks become the largest peak, at last. Although the toggle switch has more states in the 2D state space, compared to the transcription regulatory network discussed in the case study I, the time curves overlaid on the heatmap, which includes nearly thirty thousand map cells, can still provide an overview of the temporal probability distributions (Fig. 11). However, we can only see three peaks according to the curves shapes. The fourth peak is actually a spike at the location (0, 0), which can be seen in the surface plot, and as such was dismissed from the analysis.

### Informal feedback

Over multiple discussion sessions, the domain experts provided positive feedback. The junior researcher pointed out that the visual approach would be beneficial to experts working in the field when exploring the data. In particular, the lower-dimensional plots and encodings go beyond current approaches to showing the data, especially for exploring the dynamics of the system. The feature that generated most excitement for both domain experts was the ability to track the temporal changes of the probability distributions. The senior expert specifically commented on the potential of the tool to assist in deriving new hypotheses, beyond the post-hoc abilities of explanatory visualization. In addition to tracking the peak trajectories in the transcription regulatory network and the toggle switch system, the senior expert also indicated the potential use of the visual approach to systems with potentially oscillatory peak states, or to track the peak trajectory for other types of stochastic networks.

## Discussion and conclusions

The two case studies and expert feedback demonstrate that PRODIGEN is an effective visualization tool for the exploration of the probability landscape of stochastic gene regulatory networks. The system successfully allowed stochastic network researchers to perform the T1 through T4 tasks we had identified in the domain characterization stage, while handling gracefully the large scale of the data. In particular, the domain experts were able to: 1) visualize probabilities of stable states, 2) explore the temporal aspect of probability distributions, and 3) discover and analyze small peaks in the probability landscape that had potential relation to specific diseases. In both case studies they were able to identify new findings (including numerical errors) as well as replicate previous findings. The web-based implementation of the system makes it potentially available to a wide audience. Furthermore, the case studies performed with domain experts indicate the interface is user-friendly and easy to learn.

With respect to the visual design of the system, the overall design successfully supports the exploration of probability distributions in both state and time space. While some of the individual visual encodings employed are not new, the enhanced 2D heatmaps and the peak glyphs are novel contributions. The combination of visual encodings to explore data in multiple dimensions is also novel. As shown by the case studies, the visual encodings proposed were able to capture probability distributions in multiple dimensions and at multiple levels. The spaghetti plots and heatmaps overlaid by time curves are able to display the temporal probability distributions over the state space. In addition to these overviews, detail views embedded in the heatmaps provide the ability to track the probability dynamics of a user selected state. When combined together with the peak glyphs and the animated surfaces, these visual encodings satisfy the requirements from experts for detecting the number of peaks, the locations of peaks, the values of peaks, and their dynamic changes. Because the peak values vary significantly, the experts required we do not normalize axis or color; instead, we clearly indicate each scale in the interface. For example, the fourth small peak in the center plot in Fig. 12 is not perceivable when using normalization.

In terms of limitations, the system currently requires a copy of the data to be created locally and loaded for processing and visualization. PRODIGEN can only be used as a post simulation analysis tool, due to this data pre-processing load. A direction of great interest to our collaborators, although beyond the scope of this work, is the ability to run the system directly in situ, on the cluster where the simulations are computed.

In terms of scalability, the tool can handle with no issues models whose size in terms of species is on par with the ones developed by our collaborators, and was built with visual scalability in mind. Furthermore, we have tested the system on a variety of state space size and timestep configurations. As discussed in the Task Analysis section, the systems modeled typically contain few molecular species. However, the system modeled may be large in both the state space and time. Our visual approach scales well, in this respect, in either state space or time dimension. The entire system behaves well for stochastic networks with a relatively large state space size of 680430 states, which is roughly 18MB, as long as the simulation of these systems stays below several hundred timesteps. This last limitation is related to the D3 library limitations regarding loading large datasets. For practical purposes, to circumvent this limitation, simulations can be split into chunks of time and loaded and analyzed sequentially. The time-curve heat maps can further suffer from scalability issues for very large state space sizes, which can lead to small tiles and aliasing. On lower resolution displays, this issue becomes visible for large state spaces with long runs of close to a thousand timesteps, as indicated in Fig. 11. Context+focus techniques [34] may help address aliasing issues.

In conclusion, we have presented a novel web-based visual approach for the systematic exploration of probability distributions over simulation time and state space in stochastic gene regulatory networks. We provided a description of the domain data and tasks in stochastic biological network modeling and analysis, and designed a visual solution to meet the domain analysis challenges. Our visual approach combines visual encodings that consist of spaghetti plots over 1D projection, heatmaps over 2D projections, enhanced with time curves to display temporal changes, peak glyphs displayed as small multiples, and animated probability surfaces. We implemented an interactive web-based visual explorer, PRODIGEN, that combines these visual encodings to enable the exploration of probability distributions across both state and time, and we evaluated the visualization system through two regulatory networks case studies with domain experts. The case studies and the domain expert feedback indicate the effectiveness of this visual approach in helping biologists to explore the probability landscape of stochastic gene regulatory networks.

## Additional file

Additional file 1 BioVis16-121-PRODIGEN.mp4. A video demo shows how to use the application. (MP4 29901 kb)

## Abbreviations

ACME: Accurate chemical master equation
CME: Chemical master equation
